# [K_2_PbX][Ga_7_S_12_] (X = Cl, Br, I): The First Lead‐Containing Cationic Moieties with Ultrahigh Second‐Harmonic Generation and Band Gaps Exceeding the Criterion of 2.33 eV

**DOI:** 10.1002/advs.202207630

**Published:** 2023-02-27

**Authors:** Wen‐Fa Chen, Bin‐Wen Liu, Shao‐Min Pei, Xiao‐Ming Jiang, Guo‐Cong Guo

**Affiliations:** ^1^ State Key Laboratory of Structural Chemistry Fujian Institute of Research on the Structure of Matter Chinese Academy of Sciences Fuzhou Fujian 350002 P. R. China; ^2^ University of Chinese Academy of Sciences Beijing 100049 P. R. China; ^3^ Fujian Science & Technology Innovation Laboratory for Optoelectronic Information of China Fuzhou Fujian 350002 P. R. China

**Keywords:** chalcogenides, nonlinear optical materials, Pb element, second‐harmonic generation, structure–property relationship

## Abstract

In contrast to anionic group theory of nonlinear optical (NLO) materials that second‐harmonic generation (SHG) responses mainly originate from anionic groups, structural regulation on the cationic groups of salt‐inclusion chalcogenides (SICs) is performed to make them also contribute to the NLO effects. Herein, the stereochemically active lone–electron‐pair Pb^2+^ cation is first introduced to the cationic groups of NLO SICs, and the resultant [K_2_PbX][Ga_7_S_12_] (X = Cl, Br, I) are isolated via solid‐state method. The features of their three‐dimensional structures comprise highly oriented [Ga_7_S_12_]^3−^ and [K_2_PbX]^3+^ frameworks derived from AgGaS_2_, which display the largest phase‐matching SHG intensities (2.5−2.7 × AgGaS_2_ @1800 nm) among all SICs. Concurrently, three compounds manifest band gap values of 2.54, 2.49, and 2.41 eV (exceeding the criterion of 2.33 eV), which can avoid two‐photon absorption under the fundamental laser of 1064 nm, along with the relatively low anisotropy of thermal expansion coefficients, leading to improved laser‐induced damage thresholds (LIDTs) values of 2.3, 3.8, and 4.0 times that of AgGaS_2_. In addition, the density of states and SHG coefficient calculations demonstrate that the Pb^2+^ cations narrow the band gaps and benefit SHG responses.

## Graphical abstract

By introducing stereochemically active lone–electron‐pair cations in the cationic groups of salt‐inclusion chalcogenides (SICs), three compounds [K_2_PbX][Ga_7_S_12_] (X = Cl, Br, I) are successfully obtained, which display the highest phase‐matching nonlinear optical (NLO) responses in reported SICs and suitable band gaps exceeding 2.33 eV to avoid two‐photon absorption at 1064 nm.

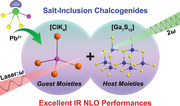

## 1 Introduction

Nonlinear optical (NLO) crystals enable the production of coherent lasers owing to their frequency conversion ability, serving as the core component of solid‐state lasers in modern optical science and technology.^[^ ^1^, ^2^, ^3^, ^4^, ^5^, ^6^, ^7^, ^8^, ^9^, ^10^, ^11^, ^12^, ^13^, ^14^, ^15^, ^16^, ^17^, ^18^ ^]^ To date, notable infrared (IR) NLO crystalline materials include commercially available AgGaQ_2_ (Q = S, Se), and ZnGeP_2_ because of their large polarizability microstructures that can lead to remarkable second‐harmonic generation (SHG) responses and superior optical transparency window in the IR range.^[^ ^19^, ^20^, ^21^ ^]^ However, they suffer from inherent deficiencies, such as the relatively low laser‐induced damage thresholds (LIDTs) of AgGaQ_2_ or the unexpected multi‐phonon absorption of ZnGeP_2_, hindering their wide application in high‐power laser field.^[^ ^22^, ^23^, ^24^, ^25^ ^]^ Therefore, an efficient route to explore IR NLO material with excellent comprehensive performance remains a challenge.

In general, the desirable characteristics for prominent IR NLO materials should be technically demanded, including large NLO coefficients, broad band gaps for high laser damage thresholds, broad transmitting spectra, moderate birefringence, and stable physical and chemical properties. Strong NLO coefficients and wide band gaps are two critically important but contradictory properties within an NLO material. From the chemical design point of view, “functional motif theory,” combining the NLO with LIDT functional motifs, has been proven to be an efficient strategy.^[^ ^26^ ^]^ Alternately, salt‐inclusion chalcogenides (SICs) [A_x_X_y_][M_m_Q_n_] (A: alkali, alkali‐earth, or rare metals; X: halogen; M: transition metal or main group; Q: chalcogen) have been synthesized and characterized by us and other researchers.^[^ ^27^, ^28^, ^29^, ^30^, ^31^, ^32^, ^33^, ^34^, ^35^, ^36^, ^37^ ^]^ Such a compound contains covalent anionic groups that enable polarization ability for remarkable SHG responses as well as strong electropositive alkali (or alkaline, rare earth) metals and electronegative halogens in cationic groups that are conducive to obtaining wide band gaps for enhancing LIDTs; examples include recently discovered [A_3_X][Ga_3_PS_8_] (A = K, Rb; X = Cl, Br),^[^ ^27^ ^]^ [ABa_2_Cl][Ga_4_S_8_] (A = Rb, Cs),^[^ ^28^ ^]^ [ABa_3_Cl_2_][Ga_5_S_10_] (A = K, Rb, Cs),^[^ ^29^ ^]^ [K_4_Cl][CdGa_9_Q_16_] (Q = S, Se),^[^ ^30^ ^]^ [K_3_Cl][Mn_2_Ga_6_S_12_],^[^ ^31^ ^]^ and [NaBa_4_Cl][Ge_3_S_10_].^[^ ^32^ ^]^ Furthermore, SICs exhibit structural features that allow the building blocks of the hosts and guests to be flexibly optimized, thereby providing infinite possibilities for NLO material design. As expected, the SIC [Ba_4_Cl_2_][HgGa_4_S_10_] with enhanced SHG intensities was derived from [Ba_4_Cl_2_][ZnGa_4_S_10_] by replacing Zn with Hg atoms in its anionic group.^[^ ^33^, ^34^ ^]^ By comparison, for tailoring the structural regulation, the incorporation of an A‐free element into the cationic group of the SIC as an NLO material has yet to be reported.

Over the last decade, introducing stereochemically active lone–electron‐pair cations, such as Pb^2+^, Sn^2+^, Bi^3+^, Sb^3+^, and As^3+^, as a result of the “additive” effect of these types of polarization, has been intensively investigated in pursuit of high NLO responses.^[^ ^38^, ^39^, ^40^ ^]^ Among these cations, Pb^2+^ makes considerable contribution to SHG response but adversely affects the band gap. However, crystals with a band gap exceeding 2.33 eV can be efficiently pumped by fundamental 1064 nm laser sources because of the absence of two‐photon absorption and free‐carrier absorption.^[^ ^41^ ^]^ Therefore, skillfully obtaining a vital band gap wider than 2.33 eV while achieving a giant SHG response in IR NLO material design becomes crucial but challenging. These excellent predictions motivated us to investigate whether we might introduce A‐free active lone‐electron‐pair cations, such as Pb^2+^, into the cationic group of SICs to generate novel IR NLO materials with the expected strong SHG intensity and band gap. Hence, comprehensive research into the K−Ga−S−PbX_2_ (X = Cl, Br, I) system, the first lead‐containing SICs, [K_2_PbX][Ga_7_S_12_] (X = Cl, **1**; Br, **2**; I, **3**), have been successfully designed and synthesized by adding lead element to the cationic groups. Intriguingly, compounds **1**−**3** have the largest SHG intensities among lead‐containing chalcogenides with band gaps greater than 2.33 eV.^[^ ^42^, ^43^, ^44^ ^]^ This host–guest system not only enhances SHG responses but also exceeds the crucial band gaps for improvement of the LIDT by involving stereochemically active lone–electron‐pair cations in SICs.

## 2 Results and Discussion

Compounds **1**−**3** were successfully grown in sealed silica tubes by high‐temperature solid‐state method. The powder X‐ray diffraction (XRD) patterns of the compounds are in good agreement with the simulated ones (Figure S1, Supporting Information), suggesting that they are pure phases. The energy‐dispersive X‐ray spectroscopy (EDS) analyses of crystals **1**−**3** verify the presence of K, Pb, Cl (or Br, I), Ga, and S and displays the molar ratio of each element well similar to chemical formula one (Figure S2, Supporting Information). Furthermore, the EDS mapping analyses demonstrate that the elements present in the compounds are equally distributed. In addition, the IR spectra demonstrate that compounds **1**−**3** have no absorptions caused by chemical bond vibrations in the IR spectra of powder samples of 2.5–25.0 µm, covering the two IR critical atmospheric transparency windows (Figure S3, Supporting Information).

**Figure 1:**
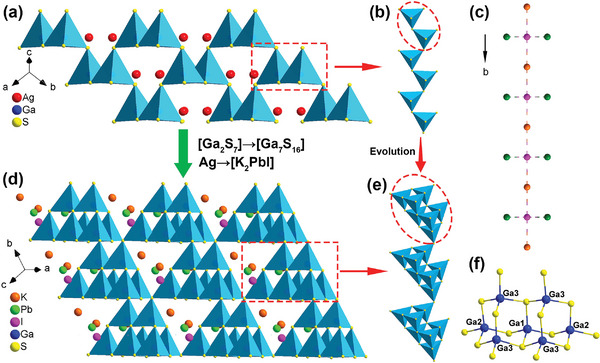
a,d) Structural evolution from AGS to compound **3** by substituting [Ga_7_S_16_] and [K_2_PbI] for [Ga_2_S_7_] and Ag units, respectively. b) 1D infinite [Ga_2_S_7_] chain. c) [K_2_PbI]^3+^ cationic group chain along the *b* axis. Green atoms, co‐occupied by Pb^2+^ and K^+^ ions with a ratio of 1:1. e) 1D infinite [Ga_7_S_16_] chain constructed from double supertetrahedral units. f) [Ga_7_S_16_]^11−^ composed of T2‐supertetrahedral by Ga(1)S_4_‐shared.

Single‐crystal XRD patterns manifest that phases **1**−**3** belong to the polar orthorhombic space group *Imm*2 (No. 44, Table S1, Supporting Information). The asymmetric unit has seven Ga atoms, 12 S atoms, one Cl (or Br, I) atom, one K atom, and one M site (that is assigned to a mixed occupancy of K/Pb with a ratio of 1:1, Table S2, Supporting Information). Compounds **1**−**3** are isostructural, and only the crystal structure of compound **3** is discussed in detail as an example. As illustrated in **Figure** **1**d, compound **3** features a three‐dimensional (3D) architecture composed of order‐accumulation [Ga_7_S_12_]^3−^ host moieties and [K_2_PbI]^3+^ guest moieties. Regarding the [Ga_7_S_12_]^3−^ polyanionic structure, the Ga(1,2,3) atoms are four‐coordinated with the sulfur atoms to create the tetrahedral [Ga(1,2,3)S_4_]^5−^ basic units with Ga—S bond lengths ranging from 2.2298(2) Å to 2.3657(1) Å (Table S3, Supporting Information). Each one Ga(1)S_4_, one Ga(2)S_4_, and two Ga(3)S_4_ are S‐vertex shared to enclose into an [Ga_4_S_10_]^8−^ T2 supertetrahedron, and two supertetrahedra further polymerize into an [Ga_7_S_16_]^11−^ double supertetrahedra (Figure 1f) by sharing one Ga(1)S_4_ tetrahedron. To the best of our knowledge, the characteristic of the [Ga_7_S_16_]^11−^ unit is first observed in SIC systems. The bridge S atoms of [Ga_7_S_16_]^11−^ double supertetrahedra serve as connectors to establish the orderly stacked 3D [Ga_7_S_12_]^3−^ host framework (Figure 1d,e). By contrast, the I^−^ anion is four coordinated by two K^+^ and two M^n+^ cations to produce a [K_2_PbI]^3+^ unit with the I—K bond distances (3.501−3.692 Å) and I—Pb bond lengths (3.027 Å), which are comparable to those of K_2_ReI_6_,^[^ ^45^ ^]^ K_2_AgI_3_,^[^ ^46^ ^]^ Pb_14_O_8_I_12_,^[^ ^47^ ^]^ and Pb_18_O_8_Cl_15_I_5_.^[^ ^38^ ^]^ Each [IK_3_Pb] tetrahedral unit gives rise to the [K_2_PbI]^3+^ line by sharing K atoms. The [K_2_PbI]^3+^ guest moiety then extends in the channels formed by the [Ga_7_S_12_]^3−^ network along the *b*‐axis direction (Figure 1c).

Moreover, the structural evolution from AGS (space group: *I*‐42*d*) to compound **3** is depicted in Figure 1. The S atoms located at the seven vertices of the [Ga_2_S_7_]^8−^ dimers in AGS as “extension sites” are filled with GaS_4_ tetrahedra in compound **3**. That is, [Ga_2_S_7_]^8−^ is substituted by a [Ga_7_S_16_]^11−^ unit. At the same time, the [K_2_PbI]^3+^ polycation group replaces Ag^+^ to build novel Pb‐containing SICs. The aforementioned evolution processes can provide additional favorable structural features. Primarily, the substitution of Ag^+^ with a [K_2_PbI]^3+^ unit breaks the all‐covalent structure of AGS and reduces the detrimental effects of photo‐sensitive Ag^+^ cations on the band gap and LIDT. According to the previously discovered SICs, such structures always exhibit the covalent host moiety and the ionic guest moiety. Pb in derivative compounds **1**−**3** does not occupy the covalent framework but rather locates the more extensive sites of cationic groups.

**Figure 2:**
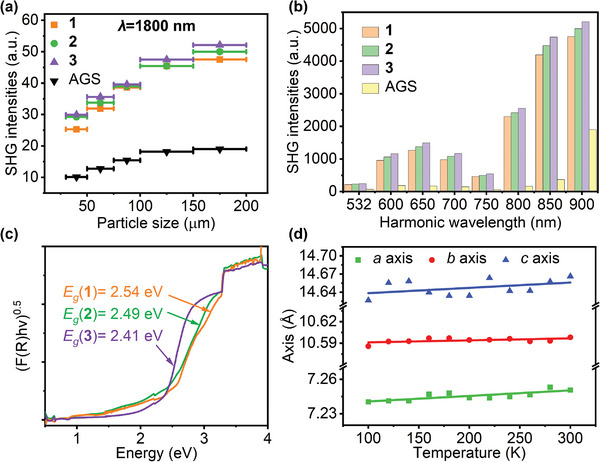
a) Phase matchable behaviors of **1**−**3** and AGS. b) Broadband SHG intensities of **1**−**3** and AGS. c) Band gaps of **1**−**3**. d) Temperature variation of the lattice parameters of **3**.

**Figure 3:**
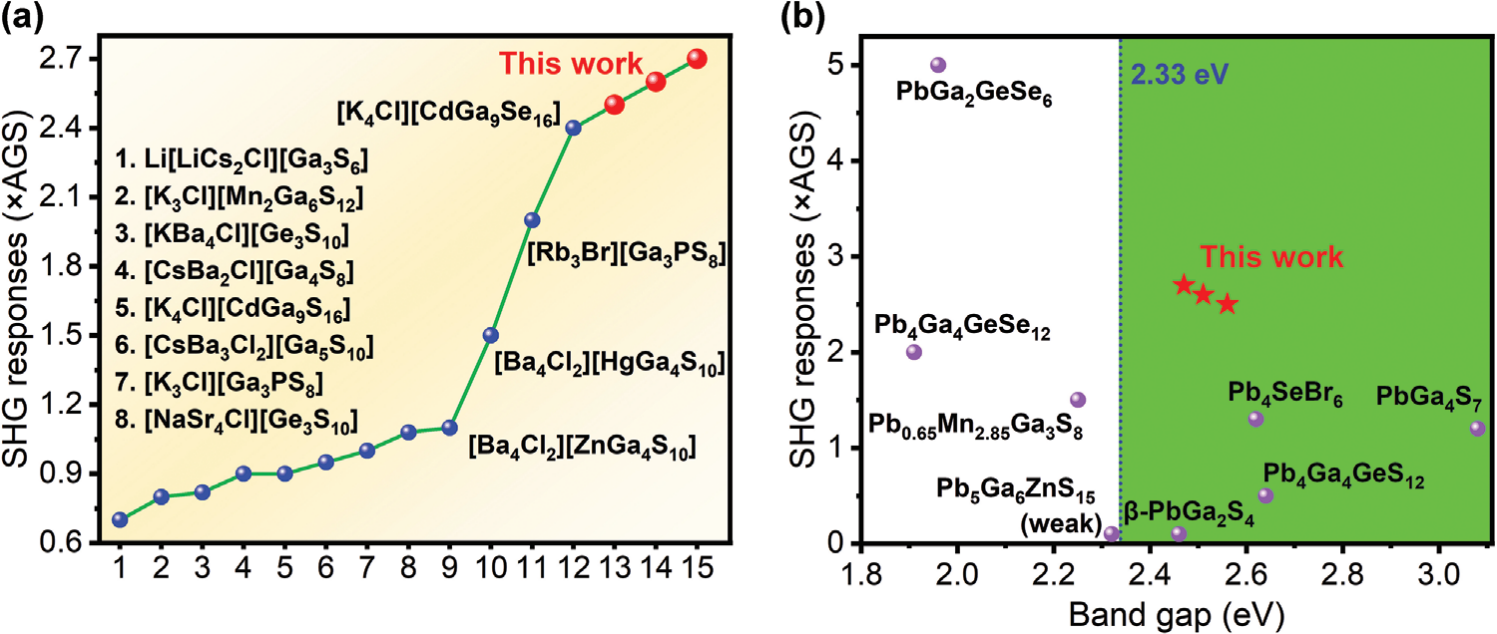
a) SHG responses for related SICs. b) Balanced performance of the SHG response and band gap for Pb‐containing NLO chalcogenides.

The powder SHG signals of **1**−**3** and benchmark AGS sieved into various sizes were measured utilizing Kurtz and Perry technique^[^ ^48^ ^]^ under different laser wavelengths of 1064−1800 nm. The results indicate that the SHG efficiencies rise with increasing particle sizes (**Figure** **2**a and Figure S5, Supporting Information), revealing type I phase‐matching behavior. As depicted in Figure 2b, in the particle size range of 150−200 µm, at 1064, 1200, 1300, 1400, 1500, 1600, 1700, and 1800 nm, the SHG intensities are 3.1, 5.2, 7.3, 6.7, 7.7, 14.1, 11.4, and 2.5 times that of AGS for **1**; 3.3, 5.8, 8.0, 7.4, 8.3, 14.8, 12.2, and 2.6 times that of AGS for **2**; and 3.6, 6.3, 8.6, 8.0, 9.0, 15.7, 12.9, and 2.7 times that of AGS for **3**. To the best of our knowledge, crystals **1**−**3** possess the largest phase‐matching SHG intensities at 1800 nm in SICs (**Figure** **3**a). Such dates are certainly larger than those in SICs Li[LiCs_2_Cl][Ga_3_S_6_] (0.7 × AGS),^[^ ^49^ ^]^ [A_3_Cl][Ga_3_PS_8_] (A = K, Rb) (1.0−1.1 × AGS),^[^ ^27^ ^]^ [ABa_2_Cl][Ga_4_S_8_] (A = Rb, Cs) (0.9−1.0 × AGS),^[^ ^28^ ^]^ and [K_3_Cl][Mn_2_Ga_6_S_12_] (0.8 × AGS).^[^ ^31^ ^]^ In addition to microscopic GaS_4_ tetrahedrons, Pb^2+^ in the cationic groups plays vital roles in the SHG intensities of **1**−**3**. The SHG efficiency order is reasonable because they are isostructural, and as the atomic weight of the halogen rises, so does the atomic contribution to the density of states close to the Fermi level, resulting in a larger SHG response. Consequently, the corresponding SHG coefficients *d*_eff_ [*d*_eff_(sample) = (*I^2^*_2_ *_*ω*_* (sample)/*I^2^_2*ω*_* (AGS))^1/2^)·*d*_eff_(AGS)] of **1**−**3** are calculated to be 14.2, 14.7, and 15.1 pm V^−1^ with the benchmark AGS (*d*_eff_ = 11.6 pm V^−1^), respectively.

For the development of high‐power laser systems, high laser‐induced damage is a critical parameter for an NLO crystal. The LIDTs of **1**−**3** were analyzed using single‐pulse method at 1064 nm,^[^ ^50^ ^]^ and AGS served as the standard. The LIDT results indicate that polycrystalline samples **1**−**3** possess comparatively large LIDTs of 2.5, 2.3, and 4.0 times that of AGS, respectively (Tables S4 and S5, Supporting Information). Based on previous investigations, crystals encounter thermal expansion, distortion, strain, catastrophic shattering, cracking, and damage as a result of the optical absorption‐induced increase in temperature.^[^ ^51^ ^]^ Among them, two‐photon or multiphoton absorption is one of the most important processes that consequently influences LIDT. Wide band gaps are generally advantageous for producing large LIDTs because they can decrease the proportion of two‐photon absorptions.^[^ ^52^ ^]^ Given the introduction of heavy Pb metal into compounds **1**−**3**, the band gaps become narrow. As shown in Figure 2c, the results deduced by the Tauc plot method^[^ ^53^ ^]^ indicate that the experimental band gaps of **1**−**3** are 2.54, 2.49, and 2.41 eV, which exceed 2.33 eV (corresponding to 532 nm) and are sufficient to prevent the negative effect of two‐photon absorption under the fundamental laser of 1064 nm. In addition, a 2D map of the SHG response and band gap properties of the reported lead‐containing chalcogenides is illustrated (Figure 3b). Compounds **1**−**3** display the highest SHG intensities in the green region with band gaps greater than 2.33 eV. Thermal expansion is another phenomenon that affects LIDT. Temperature‐dependent lattice parameters were used to study the thermal expansion coefficients (TECs) of phases **1**−**3**. As shown in Table S4 (Supporting Information) and Figure 2d and Figure S5 (Supporting Information), the thermal expansion anisotropy (TEA, *δ*; defined as *δ* = max{(*α*_i_−*α*_j_)/*α*_i(i,j = a,b,c)_}) values of **1**, **2**, and **3** are 1.65, 2.27, and 1.58, respectively, which are smaller than that of AGS (2.95).^[^ ^29^ ^]^ Thus, crystals with lower thermal expansion anisotropy values and exceeding vital band gaps are more likely to present higher LIDTs.

**Figure 4:**
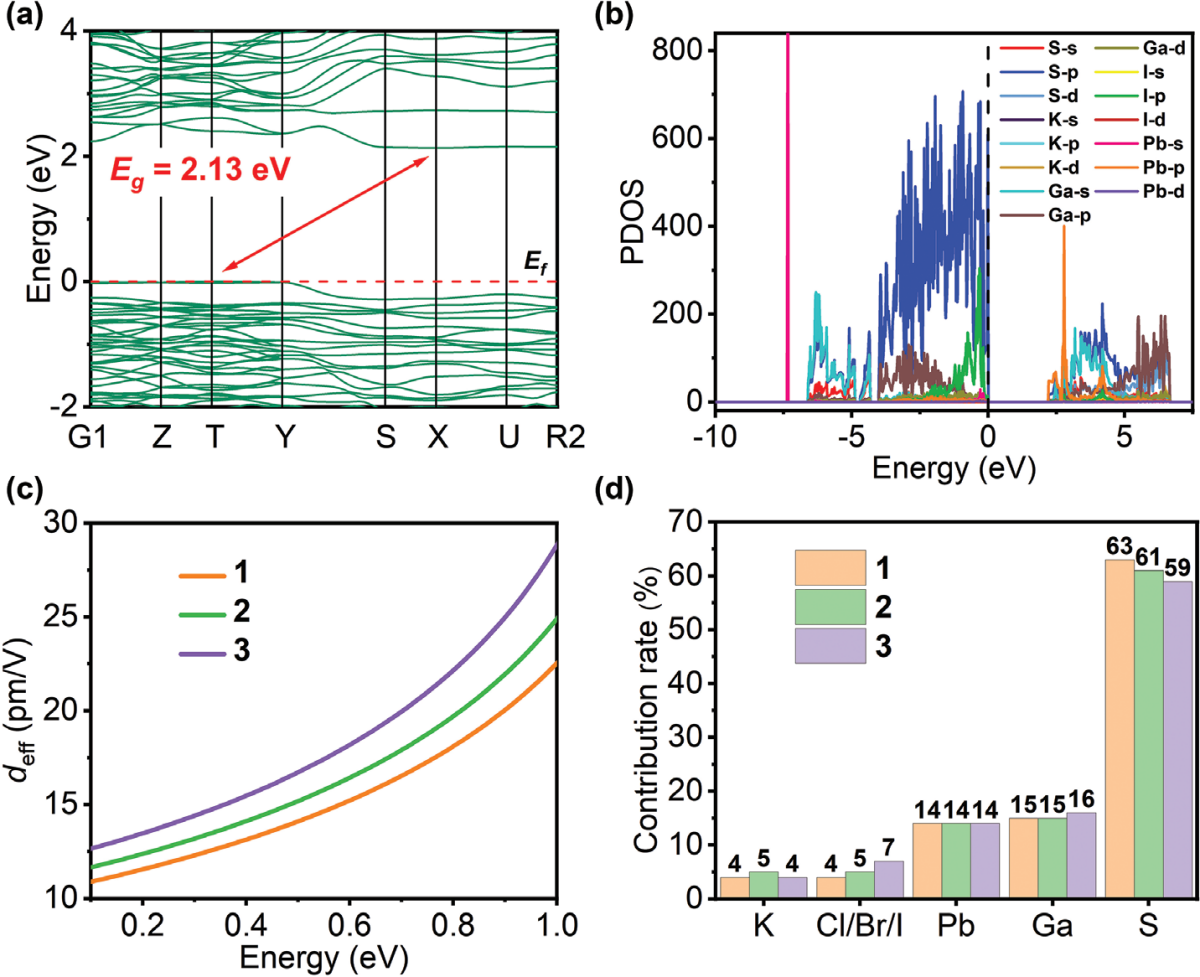
a) Calculated electronic band structure of **3**. b) PDOS of **3**. c) Calculated effective SHG coefficients of **1**−**3**, d) NLO efficiency contributions of individual atoms of **1**−**3**.

The electronic band structures were theoretically calculated to understand further the relationship between the structures and optical properties of **1**−**3**. As illustrated in **Figure** **4**a and Figure S6 (Supporting Information), the features of band structures and density of states (DOS) in compounds **1**−**3** are extremely similar: all three are indirect band gap semiconductors with valence band maximum at T point and conduction band minimum at X point. They possess the Perdew–Burke–Ernzerhof (PBE)‐calculated band gaps of 2.12, 2.10, and 2.13 eV for **1**−**3**, respectively, which are acceptable because PBE methods underestimate the band gap. As depicted in Figure 4b and Figure S7 (Supporting Information), the calculated partial density of states (PDOS) reveals that the valence band top (from −4 to 0 eV) largely comes from Ga‐4p and S‐3p mixed states and Cl‐3p/Br‐4p/I‐5p states, whereas the conduction band bottom (from 2 to 4 eV) to a large extent consists of unoccupied Pb‐6p, S‐3p, and Ga‐4s states. The K‐3p state is located at 5–7 eV, which is far from the forbidden band edge. Therefore, the SHG responses are not only derived from the [Ga_7_S_12_]^3−^ anionic framework but also associated with the [K_2_PbX]^3+^ cationic groups because the optical properties are predominantly related to the electronic transitions around the forbidden band edge. This conclusion is quite different from previous findings that the SHG effects were primarily generated by the host moieties and the guest moieties contributed negligibly.

The NLO coefficients were calculated on the basis of the first‐principle calculation. The space groups of **1**−**3** belong to the point group of class *mm*2. Moreover, according to Kleinman's symmetry restriction, compounds **1**−**3** only have three non‐vanishing independent SHG tensors (*d*_113_ = *d*_311_, *d*_223_ = *d*_322_, *d*_333_). As the energy‐dependent SHG coefficients of **1**−**3** are displayed in Figure S8 (Supporting Information), the calculated values of *d*_113_, *d*_223_, and *d*_333_ are 20.2, −21.3, −7.5 pm V^−1^ for **1**, 21.6, −23.1, −9.3 pm V^−1^ for **2**, and 21.9, −26.2, −13.4 pm V^−1^ for **3**, respectively, under the energy of 0.69 eV (1800 nm). As depicted in Figure 4c, the calculated effective SHG coefficient *d*_eff_ values of **1**−**3** are 16.3, 17.7, and 19.7 pm V^−1^, respectively, at 1800 nm, which are slightly larger than the experimental results of 14.2, 14.7, and 15.1 pm V^−1^. Through orbital‐ and atom‐resolved SHG coefficient calculations, the atomic NLO efficiency contributions of **1**−**3** are demonstrated in Figure 4d; the S, Ga, and Pb atoms of **1**−**3** contribute considerably to the largest tensors of *d*_223_, and the contributions of the K atom to the SHG effects are negligible. Among them, the SHG contribution of Pb atoms is comparable with that of Ga atoms. The results suggest that the [Ga_7_S_12_]^3−^ host moieties dominate the macroscopic SHG efficiencies at 78%, 76%, and 75% for **1**, **2**, and **3**, respectively, whereas the [K_2_PbX]^3+^ cationic group contribute more than 20% to the SHG responses due to the introduction of Pb^2+^ ions. The Pb‐6p state occupies the bottom of the conduction band to narrow the band gaps (still above 2.33 eV) but considerably contributes to SHG responses. These results align with those of the PDOS analysis.

## 3 Conclusion

In summary, by involving the Pb in polar salt‐inclusion chalcogenides for the first time, [K_2_PbX][Ga_7_S_12_] (X = Cl, **1**; Br, **2**; I, **3**) are successfully prepared via high‐temperature solid‐state methods. The 3D frameworks are composed of tetrahedral‐packing [Ga_7_S_12_]^3−^ host and [K_2_PbX]^3+^ guest moieties derived from AGS. Remarkably, compounds **1**−**3** exhibit the highest phase‐matching SHG responses (2.5−2.7 × AgGaS_2_ @1800 nm) in reported SICs and suitable band gaps (2.41−2.54 eV) exceeding 2.33 eV to avoid two‐photon absorption at 1064 nm for high LIDTs (2.3−4.0 × AgGaS_2_) by introducing stereochemically active lone–electron‐pair Pb^2+^ in their structures. The theoretical calculations reveal that the origins of SHG effects are associated not only with the host moieties [Ga_7_S_12_]^3−^ but also with the guest moieties [K_2_PbX]^3+^ (contribution rates > 20%); this finding differs from previous understandings of the guest moieties. Hence, this research provides a new paradigm for designing and developing novel IR NLO materials with pronounced comprehensive properties.

## 4 Experimental Section

### Syntheses

All reactants were stored and processed in a glove box filled with high‐purity Argon (99.999%). For synthesis of crystals [K_2_PbX][Ga_7_S_12_] (X = Cl, **1**; Br, **2**; I, **3**), the mixtures of K metal (0.81 mmol, Sinopharm, 99%), Ga metal (2.01 mmol, Alfa, 99.99%), S powder (3.62 mmol, Sinopharm, 99.9%), and PbCl_2_/PbBr_2_/PbI_2_ powder (0.40 mmol, Aladdin, 99%) for **1**/**2**/**3**, respectively, were weighed and carefully loaded into 11‐mm (inner diameter) quartz tubes. These tubes were flame‐sealed under a vacuum of 10^−4^ Torr and then placed in the furnace. These vessels were gradually heated at room temperature to 930 °C over 25 h, maintained within 96 h, and cooled to 330 °C at a rate of 3 °C h^−1^ before they reached ambient temperature naturally. The colors of crystals **1**−**3** are bright yellow, yellow, and orange, which are mechanically separated from the products that are washed with distilled water and ethanol. All three compounds had unchanged weight and color at ambient temperature for half a year which account for their physical and chemical stability. In addition, a Hitachi S‐3500 SEM spectrometer equipped with an EDS was used to carry out the EDS element analyses of **1**−**3**.

### Single‐Crystal and Powder XRD Analysis

The crystal structures of high‐quality **1**−**3** were collected using a Rigaku FR‐X Microfocus diffractometer (45 kV, 66 mA) fitted with Mo‐K*α* radiation (*λ* = 0.71073 Å). CrysAlisPro software was used to collect and reduce data,^[^ ^54^ ^]^ and the absorption corrections based on the multi‐scan method were performed. The crystal structures were solved using the direct method and refined on *F* ^2^ by full‐matrix least squares methods through the Siemens SHELXTL crystallography software package.^[^ ^55^ ^]^ The anisotropic thermal parameters and a secondary extinction correction were applied to refine all atoms. The space groups of structures were further verified for higher symmetry by PLATON, and none was found.^[^ ^56^ ^]^ Powder XRD patterns of **1**−**3** were recorded using an automatic Rigaku Flex600 X‐ray diffractometer equipped with Cu K*α* (*λ* = 1.54057 Å) radiation in the range of 2*θ* = 5−65° with a scan step width of 0.02° at room temperature.

### IR and UV−Vis−NIR Diffuse‐Reflectance Spectroscopy

The IR spectra of powder samples **1**−**3** were determined using a Fourier transform IR spectrometer in the range of 4000−400 cm^−1^ (corresponding to the wavelength range from 2.5 to 25.0 µm). Data for the UV–vis‐near IR diffuse‐reflectance spectra were recorded with a Perkin‐Elmer Lambda 950 UV–vis–NIR spectrophotometer in the 200−2500 nm wavelength range.

### SHG and LIDT Measurements

Kurtz–Perry method was applied to perform broadband powder SHG signals at diverse lasers from 1064 to 1800 nm.^[^ ^48^ ^]^ Given that the SHG intensity is dependent on the particle size, crystalline **1**−**3** and AgGaS_2_ were ground and sieved into several particle sizes (30−50, 50−75, 75−100, 100−150, and 150−200 µm) and pressed into disks with a diameter of 6.0 mm. Moreover, LIDTs of **1**−**3** and AgGaS_2_ (particle size: 150−200 µm) were recorded by applying the single‐pulse measuring method using laser of 1064 nm wavelength (frequency, 1 Hz; pulse width, 10 ns). When the laser power gradually increased, the damaged spot on the sample was observed by an optical microscope. The LIDT values were calculated under the equation LIDT = *E*/*πr* ^2^ *τ*_p_, where *E* is the energy of a single pulse, *r* is the spot radius, and *τ*_p_ is the pulse width.

### Computational Methods

The electronic band structures, DOS, and optical characteristics of **1**−**3** were calculated using the ABINIT package based on density functional theory to understand comprehensively the relationship between structure and NLO performance.^[^ ^57^, ^58^, ^59^, ^60^ ^]^ Since the disorder in the structure was not favorable to theoretical calculations, the unit cell was expanded to generate a supercell structure, which was then used for simulation calculations. Exchange and correlation effects were treated by generalized gradient approximation. The cutoff energy was set at 18 Hartree. The following orbitals were regarded as valence electrons: K‐3s^2^3p^6^4s^1^, Pb‐5d^10^6s^2^6p^2^, Cl‐3s^2^3p^5^, Br‐4s^2^4p^5^, I‐5s^2^5p^5^, Ga‐3d^10^4s^2^4p^1^, and S‐3s^2^3p^4^. The *k* integration over the Brillouin zone was implemented by the tetrahedron method using 4 × 4 × 4 Monkhorst–Pack *κ*‐point meshes for primitive lattices of compounds **1**−**3**. Optical properties were calculated in terms of the complex dielectric function.^[^ ^61^ ^]^ The theory of density functional perturbation and the “sum over states” method served to calculate the frequency‐dependent SHG susceptibility tensor *χ*_ijk_(2*ω*,*ω*, *ω*).^[^ ^62^ ^]^ The polycrystalline effective NLO coefficient *d*_eff_ was determined by angularly averaging effective NLO coefficient tensors. The orbital‐resolved SHG susceptibility tensors were assessed using the normalized partial DOS of the participating orbitals, which satisfied the constraint that the total contributions from all involved orbitals to each term must equal 100%. The atomic‐resolved NLO susceptibility tensors were determined by summing all of the contributions from the orbitals of the target atom.

[Further details of the crystal structure investigation(s) may be obtained from the Fachinformationszentrum Karlsruhe, 76344 Eggenstein‐Leopoldshafen (Germany), on quoting the depository number CSD 2156011−2156013].

## Conflict of Interest

The authors declare no conflict of interest.

## Supporting information

Supporting Information

## Data Availability

The data that support the findings of this study are available from the corresponding author upon reasonable request.
