# Supplementary material for: [K2PbX][Ga7S12] (X = Cl, Br, I): The First Lead‐Containing Cationic Moieties with Ultrahigh Second‐Harmonic Generation and Band Gaps Exceeding the Criterion of 2.33 eV
Source: Adv Sci (Weinh). 2023 Feb 27;10(13):2207630. doi: 10.1002/advs.202207630 (PMC10161116; doi:10.1002/advs.202207630)
Supplement: Supplementary file 1 — Supporting Information [file ADVS-10-2207630-s001.pdf]

ADVANCED SCIENCE
Open Access

Supporting Information

for *Adv. Sci.*, DOI 10.1002/advs.202207630

$[K_2PbX][Ga_7S_{12}]$ (X = Cl, Br, I): The First Lead-Containing Cationic Moieties with Ultrahigh Second-Harmonic Generation and Band Gaps Exceeding the Criterion of 2.33 eV

Wen-Fa Chen, Bin-Wen Liu, Shao-Min Pei, Xiao-Ming Jiang and Guo-Cong Guo**

Supporting Information

$[K_2PbX][Ga_7S_{12}]$ (X = Cl, Br, I): The First Lead-Containing Cationic Moieties with Ultrahigh Second-Harmonic Generation and Band Gaps Exceeding the Criterion of 2.33 eV

Wen-Fa Chen,[a,b] Bin-Wen Liu,[a,c,*] Shao-Min Pei,[a,b] Xiao-Ming Jiang,[a,c] and Guo-Cong Guo[a,c,*]

[a] *State Key Laboratory of Structural Chemistry, Fujian Institute of Research on the Structure of Matter, Chinese Academy of Sciences, Fuzhou, Fujian 350002, People's Republic of China*

[b] *University of Chinese Academy of Sciences, Beijing 100049, People's Republic of China*

[c] *Fujian Science & Technology Innovation Laboratory for Optoelectronic Information of China, Fuzhou, Fujian 350108, People's Republic of China*

Table of Contents

Figures and Tables
Figure S1. Experimental and simulated powder XRD patterns of compounds **1−3**.
Figure S2. EDS results for **1−3**.
Figure S3. Infrared transmittance spectra of **1−3**.
Figure S4. The temperature-dependent lattice parameters of **1** and **2**.
Figure S5. Phase matchable behaviors of compounds **1-3** and $AgGaS_2$ at broadband wavelengths.
Figure S6. Calculated electronic band structures of **1** and **2**.
Figure S7. PDOS of **1** and **2**.
Figure S8. Calculated energy-dependent SHG coefficient tensors of **1−3**.
Table S1. Crystal data and structure refinement parameters for **1−3**.
Table S2. Fractional atomic coordinates and equivalent isotropic displacement parameters for **1−3**.
Table S3. Bond distances for **1−3**.
Table S4. The LIDT results of **1**, **2**, **3**, and $AgGaS_2$.

Figures and Tables

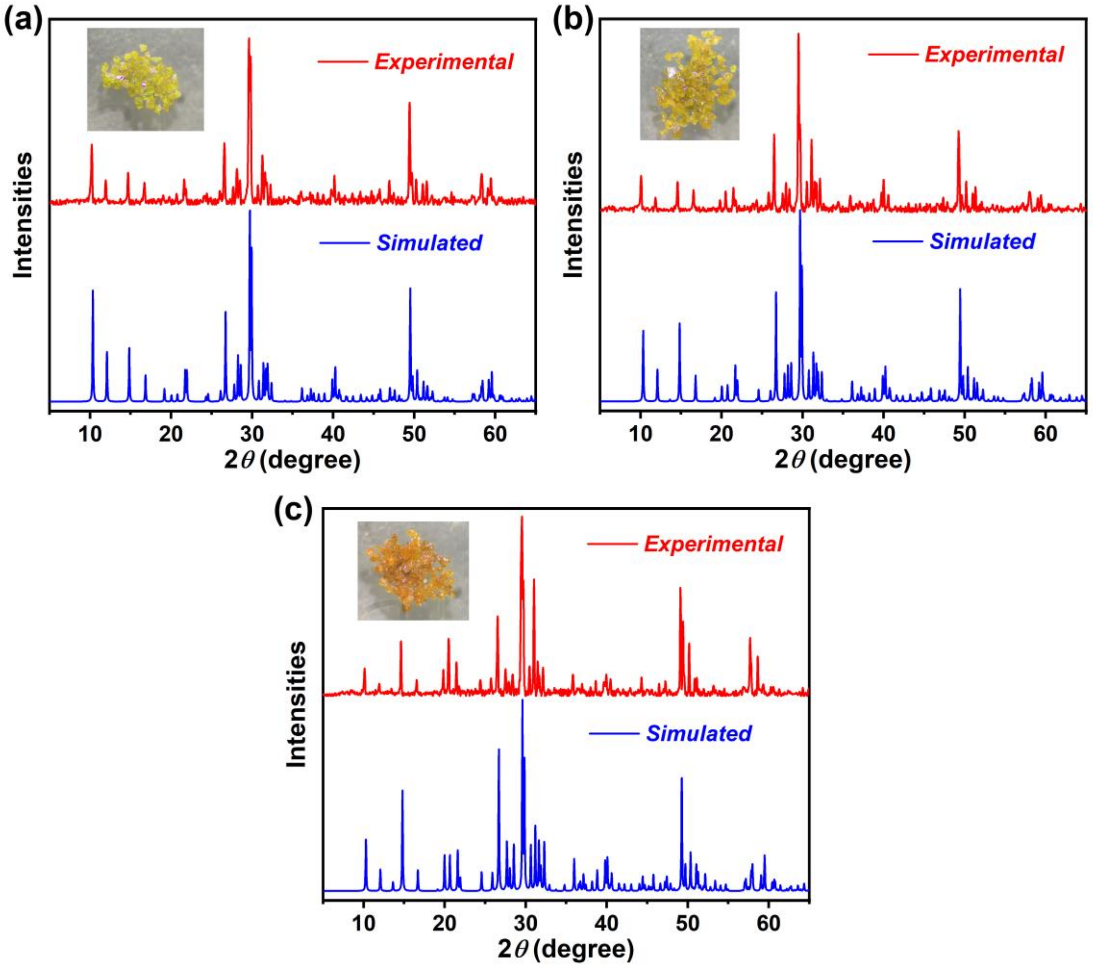

Figure S1. Experimental and simulated powder X-ray diffraction patterns of compounds **1**−**3** (a−c). Insets are the images of crystals **1**−**3**.

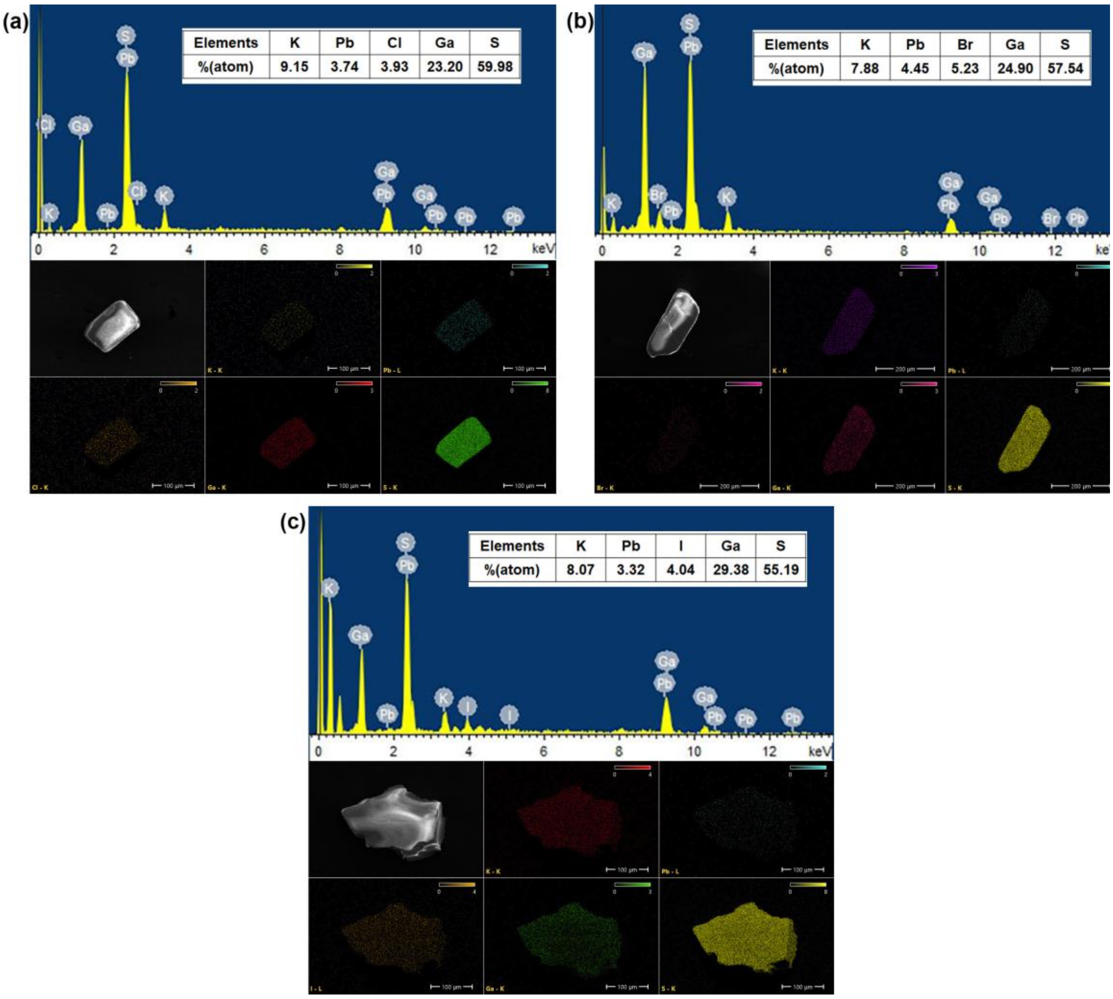

Figure S2. EDS results for **1**−**3** (a−c).

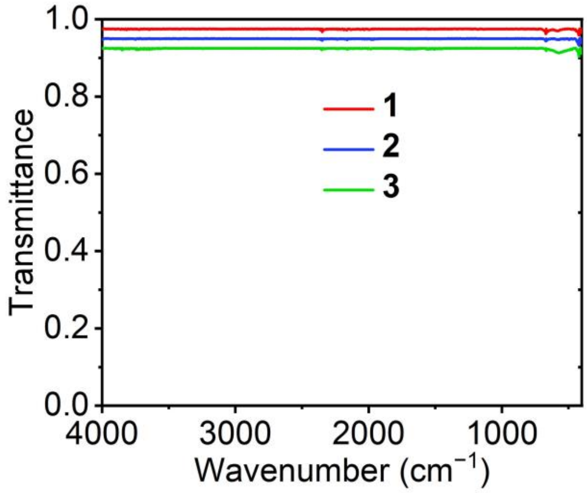

Figure S3. Infrared transmittance spectra of **1**−**3**.

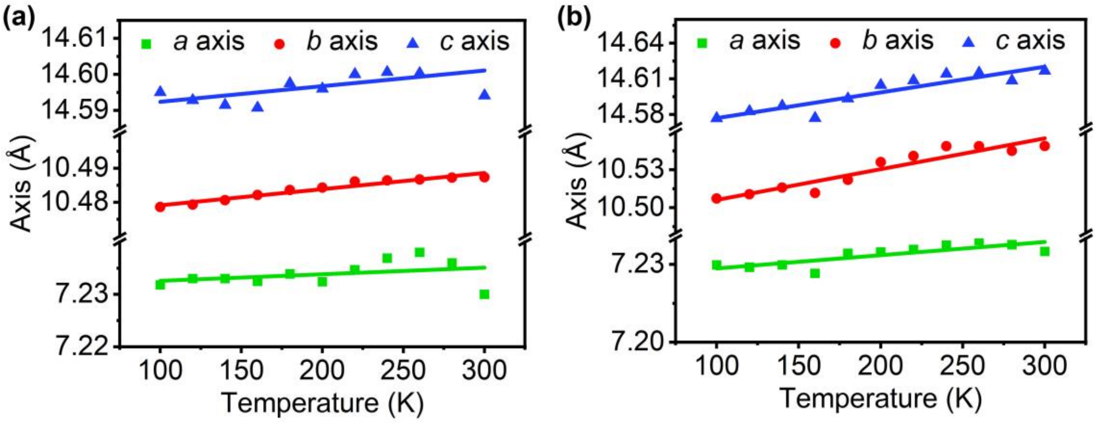

Figure S4. The temperature-dependent lattice parameters of **1** (a) and **2** (b).

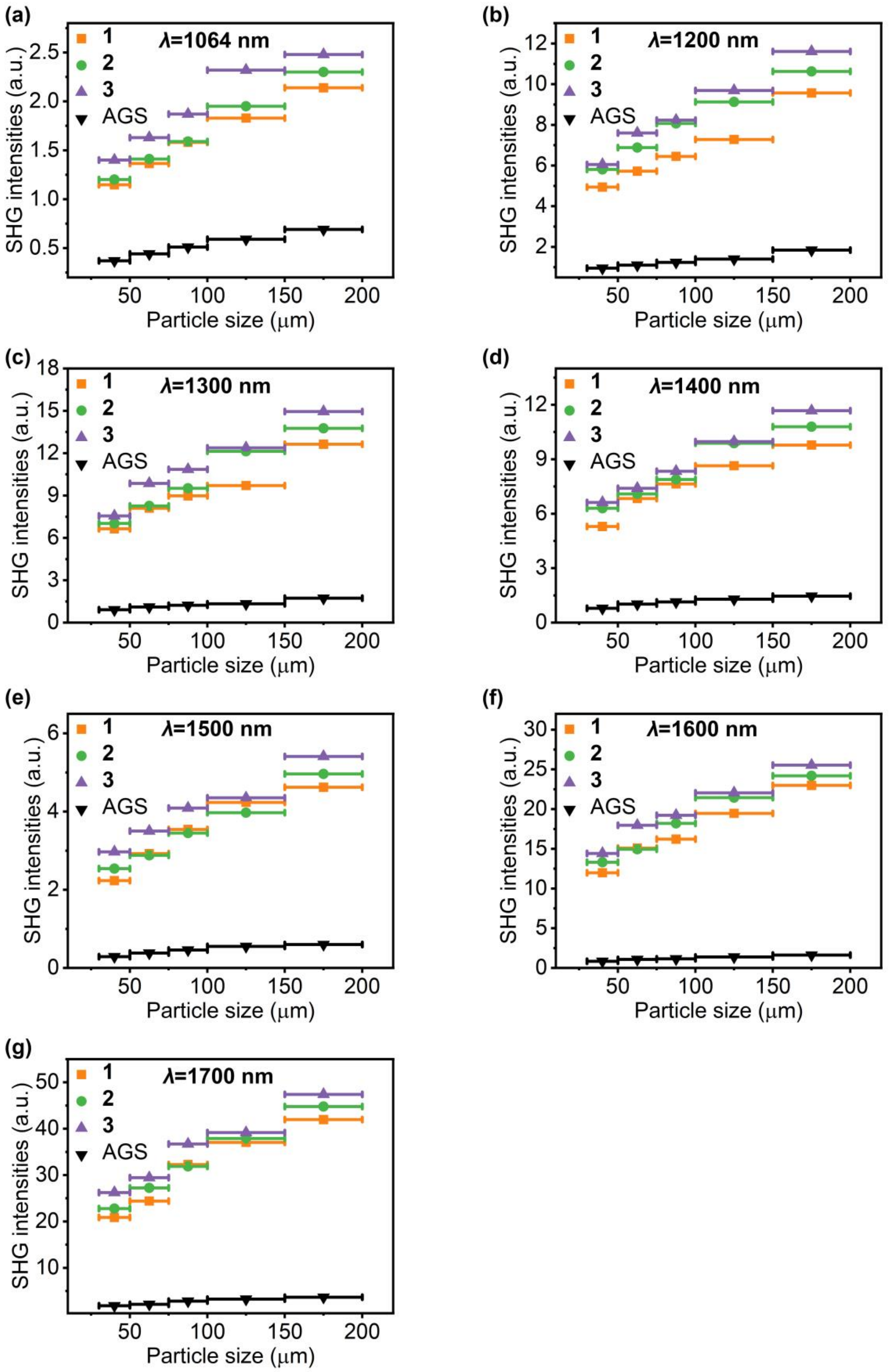

Figure S5. Phase matchable behaviors of compounds **1**−**3** and $AgGaS_2$ at broadband wavelengths.

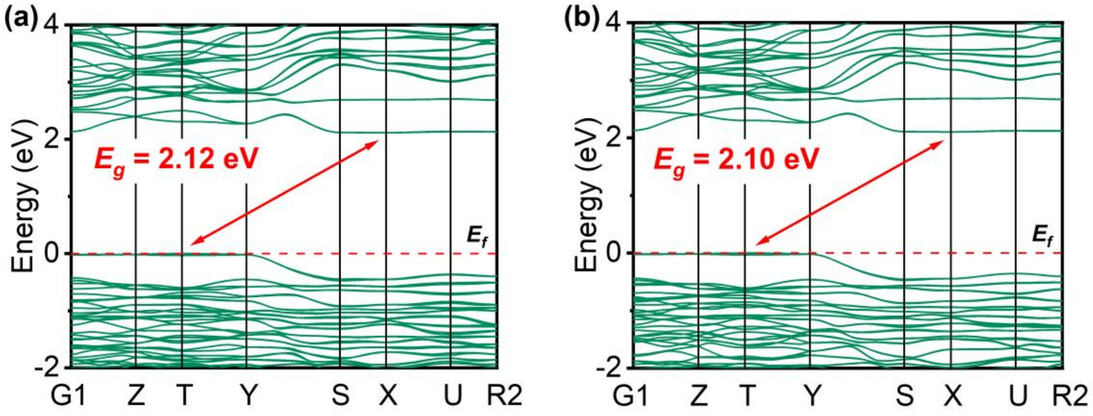

Figure S6. Calculated electronic band structures of **1** (a) and **2** (b).

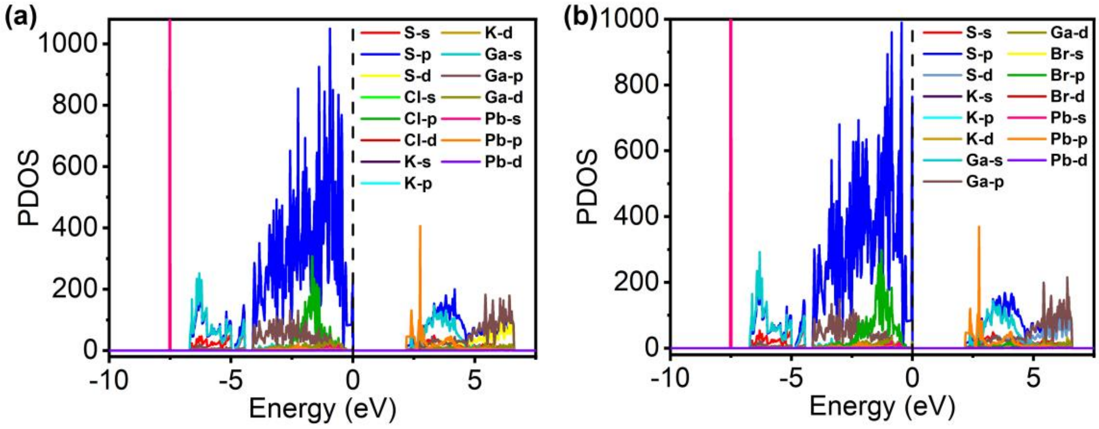

Figure S7. Partial density of states (PDOS) of **1** (a) and **2** (b).

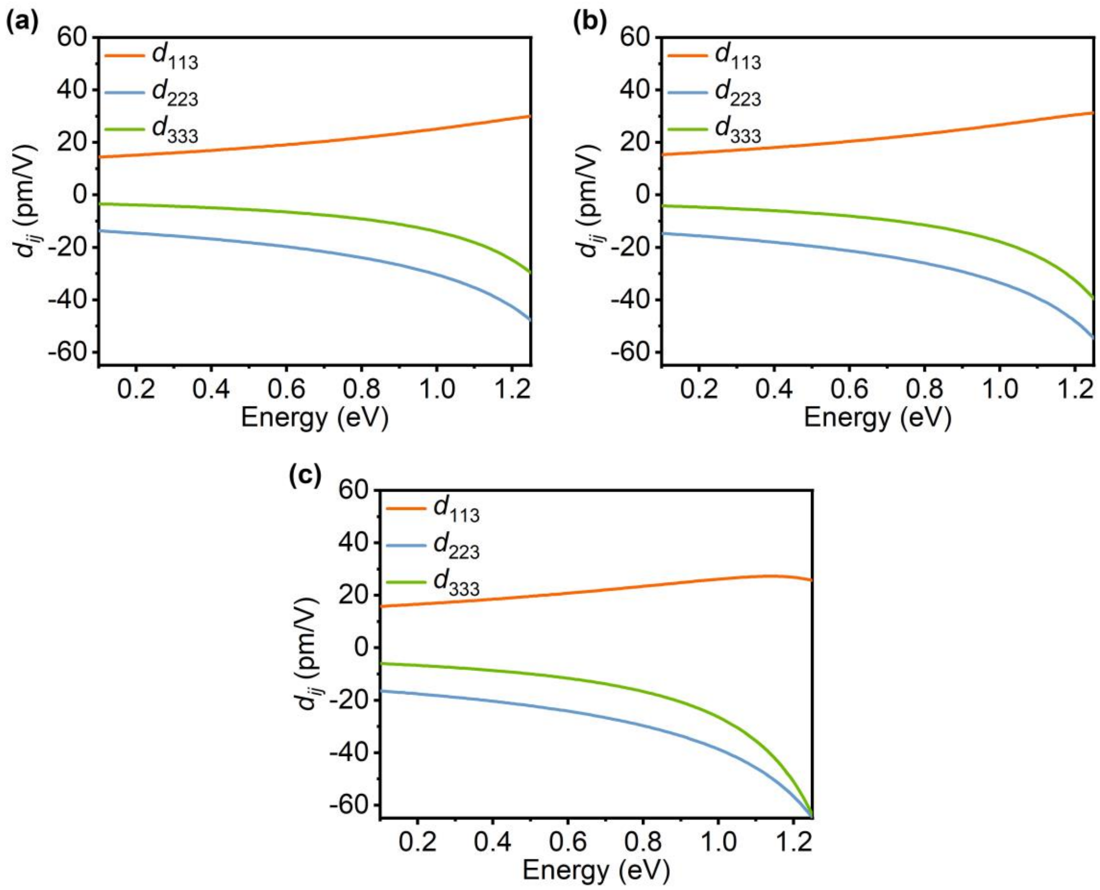

Figure S8. Calculated energy-dependent SHG coefficient tensors of **1**−**3** (a−c).

Table S1. Crystal data and structure refinement parameters for **1**−**3**.

Empirical formula	$[K_2PbCl][Ga_7S_{12}]$	$[K_2PbBr][Ga_7S_{12}]$	$[K_2PbI][Ga_7S_{12}]$
CSD number	2156011	2156012	2156013
Formula weight	1193.60	1252.14	1303.83
Temperature/K	293(2)	293(2)	293(2)
Space group	*Imm*2	*Imm*2	*Imm*2
a/Å	7.2409(3)	7.2381(3)	7.2434(3)
b/Å	10.5188(5)	10.5494(6)	10.6122(3)
c/Å	14.6351(5)	14.6346(7)	14.6625(5)
α/°	90	90	90
β/°	90	90	90
γ/°	90	90	90
Volume/Å^3	1114.69(8)	1117.46(10)	1127.08(7)
Z	2	2	2
ρ_{calc} g/cm^3	3.556	3.679	3.787
μ/mm^{-1}	17.451	19.076	18.510
GOF on F^2	1.049	0.999	1.069
$R_1{}^a$ [I>=2σ (I)]	0.0381	0.0295	0.0285
$wR_2{}^b$ [I>=2σ (I)]	0.1015	0.0733	0.0638
$R_1{}^a$ [all data]	0.0385	0.0301	0.0294
$wR_2{}^b$ [all data]	0.1018	0.0740	0.0644
Flack	0.0004(2)	0.0003(3)	-0.0001(1)
$\Delta\rho_{max}/\Delta\rho_{min}$/e Å^{-3}	1.39/-1.29	1.37/-1.65	1.59/-1.90

$^aR = \Sigma||F_o|-|F_c||/\Sigma|F_o|$, $^bwR = (\Sigma(w(F_o{}^2-F_c{}^2)^2)/\Sigma(w(F_o{}^2)^2))^{1/2}$.

Table S2. Fractional atomic coordinates (×10^4) and equivalent isotropic displacement parameters (Å^2×10^3) for **1**−**3**. U_{eq} is defined as 1/3 of the trace of the orthogonalized U_{IJ} tensor.

		**1**		
Atom	*x*	*y*	*z*	U_{eq}
K1	5000	-4180(20)	-380(8)	82(5)
K2	3340(10)	0	-3226(10)	58(4)
Pb1	3380.4(14)	0	-2597(3)	87.3(10)
Cl1	5000	0	-1209(9)	83(3)
Ga1	0	0	826(2)	13.3(4)
Ga2	2433.5(10)	0	769.7(15)	14.5(3)
Ga3	1304.4(7)	2440.8(15)	-1651.5(10)	14.0(3)
S1	0	2671(4)	-378(3)	13.8(7)
S2	1223(2)	0	2103(4)	27.2(9)
S3	3770(2)	0	2033(3)	12.5(6)
S4	2555(2)	2582(4)	-413(3)	26.7(6)
S5	1288(2)	0	-2966(3)	17.7(7)
		**2**		
Atom	*x*	*y*	*z*	U_{eq}
K1	5000	-4490(20)	-369(7)	74(6)
K2	3344(6)	0	-3255(7)	57(3)
Pb1	3363.5(12)	0	-2590(3)	91.4(9)
Br1	5000	0	-1091(3)	65.0(9)
Ga1	0	0	843.1(17)	13.4(4)
Ga2	2439.6(9)	0	790.2(14)	14.0(3)
Ga3	1306.8(6)	2443.3(13)	-1623.5(10)	14.1(2)
S1	0	2674(4)	-361(3)	12.9(6)
S2	1226(2)	0	2103(3)	27.7(7)
S3	3767.2(17)	0	2064(3)	12.3(5)
S4	2558.2(17)	2580(3)	-395(3)	25.9(5)
S5	1285(2)	0	-2935(3)	17.3(6)
		**3**		
Atom	*x*	*y*	*z*	U_{eq}
K2	5000	-5000	-417(6)	74(2)
K1	3325(4)	0	-3444(5)	34.3(18)
Pb1	3326.1(14)	0	-2763(3)	106.8(11)
I1	5000	0	-1093.8(16)	48.2(5)
Ga1	0	0	802.0(17)	12.5(4)
Ga2	2439.2(9)	0	748.9(13)	13.4(3)
Ga3	1310.6(6)	2443.7(14)	-1652.8(8)	13.8(2)
S1	0	2666(4)	-403(3)	12.8(6)
S2	1225(2)	0	2047(3)	30.0(9)
S3	3751.9(18)	0	2045(3)	11.6(6)
S4	2552.1(17)	2579(4)	-424(3)	26.0(5)
S5	1280(2)	0	-2956(3)	19.0(6)

Table S3. Bond distances for **1**−**3**.

Bond	Distance/Å	Bond	Distance/Å	Bond	Distance/Å
			**1**		
K(1)−K(1)	1.19(3)	K(2)−S(4)	3.174(11)	Ga(1)−S(2)	2.238(4)
K(1)−Cl(1)	3.149(15)	K(2)−Cl(1)	3.225(15)	Ga(1)−S(1)	2.311(3)
K(1)−S(5)	3.218(8)	K(2)−S(4)	3.684(10)	Ga(1)−S(1)	2.311(3)
K(1)−S(5)	3.218(8)	K(2)−S(4)	3.684(10)	Ga(2)−S(4)	2.253(3)
K(1)−S(2)	3.251(8)	K(2)−S(2)	3.693(3)	Ga(2)−S(4)	2.253(3)
K(1)−S(2)	3.251(8)	K(2)−S(2)	3.693(3)	Ga(2)−S(2)	2.259(4)
K(1)−S(4)	3.760(6)	K(2)−S(1)	3.724(11)	Ga(2)−S(3)	2.364(3)
K(1)−S(4)	3.760(6)	K(2)−S(1)	3.724(11)	Ga(3)−S(5)	2.244(2)
K(2)−Pb(1)	0.664(9)	Pb(1)−Cl(1)	2.784(5)	Ga(3)−S(4)	2.249(3)
K(2)−S(5)	3.015(15)	Pb(1)−S(5)	3.087(4)	Ga(3)−S(39)	2.315(2)
K(2)−S(4)	3.174(11)	Ga(1)−S(2)	2.238(4)	Ga(3)−S(1)	2.338(2)
			**2**		
Bond	Distance/Å	Bond	Distance/Å	Bond	Distance/Å
K(1)−K(1)	0.74(3)	K(2)−S(2)	3.6926(18)	Ga(1)−S(1)	2.315(3)
K(1)−S(5)	3.205(7)	K(2)−S(2)	3.6926(18)	Ga(1)−S(1)	2.315(3)
K(1)−S(5)	3.205(7)	K(2)−S(1)	3.694(7)	Ga(2)−S(2)	2.252(3)
K(1)−S(2)	3.236(7)	K(2)−S(1)	3.694(7)	Ga(2)−S(4)	2.254(2)
K(1)−S(2)	3.236(7)	K(2)−S(4)	3.730(7)	Ga(2)−S(4)	2.254(2)
K(1)−Br(1)	3.336(14)	K(2)−S(4)	3.730(7)	Ga(2)−S(3)	2.362(3)
K(2)−Pb(1)	0.702(6)	Pb(1)−Br(1)	2.870(3)	Ga(3)−S(5)	2.245(2)
K(2)−S(5)	3.032(9)	Pb(1)−S(5)	3.064(4)	Ga(3)−S(4)	2.246(3)
K(2)−S(4)	3.148(7)	Ga(1)−S(2)	2.233(3)	Ga(3)−S(3)	2.3140(19)
K(2)−S(4)	3.148(7)	Ga(1)−S(2)	2.233(3)	Ga(3)−S(1)	2.3366(19)
K(2)−Br(1)	3.329(8)				
			**3**		
Bond	Distance/Å	Bond	Distance/Å	Bond	Distance/Å
K(2)−S(5)	3.216(6)	K(1)−S(5)	3.043(7)	Ga(1)−S(1)	2.316(3)
K(2)−S(5)	3.216(6)	K(1)−I(1)	3.501(5)	Ga(2)−S(2)	2.251(4)
K(2)−S(2)	3.236(6)	K(1)−S(1)	3.635(5)	Ga(2)−S(4)	2.251(3)
K(2)−S(2)	3.236(6)	K(1)−S(1)	3.635(5)	Ga(2)−S(4)	2.251(3)
K(2)−I(1)	3.6923(12)	K(1)−S(2)	3.7180(14)	Ga(2)−S(3)	2.366(3)
K(2)−I(1)	3.6923(12)	K(1)−S(2)	3.7180(14)	Ga(3)−S(4)	2.241(3)
K(1)−Pb(1)	0.723(4)	Pb(1)−S(5)	3.007(4)	Ga(3)−S(5)	2.247(2)
K(1)−S(4)	3.024(5)	Pb(1)−I(1)	3.027(3)	Ga(3)−S(3)	2.312(2)
K(1)−S(4)	3.024(5)	Ga(1)−S(1)	2.316(3)	Ga(3)−S(1)	2.3405(19)

Table S4. Comparison of LIDTs, band gaps, TECs, and TEA of **1**, **2**, **3**, and $AgGaS_2$.

Compounds	LIDT (MW/cm^2)	Band gap (eV)	TEC ($\times 10^{-5}$)			TEA
			a	*b*	*c*	
**1**	9.45	2.56	0.17	0.32	0.30	1.65
**2**	8.46	2.51	0.71	2.32	1.48	2.27
**3**	14.9	2.47	0.67	0.26	0.60	1.58
$AgGaS_2$	3.73	2.62	2.09	2.09	−1.07	2.95

Table S5. The LIDT results of **1**, **2**, **3**, and $AgGaS_2$.

Compounds	Damage energy (mJ)	Spot area (cm^2)	τ_p (ns)	Damage threshold [$MW\cdot cm^{-2}$]
**1**	1.9	0.02	10	9.4
**2**	1.7	0.02	10	8.5
**3**	3.0	0.02	10	14.9
$AgGaS_2$	0.75	0.02	10	3.7
